# Advances in Urinary Diversion: From Cutaneous Ureterostomy to Orthotopic Neobladder Reconstruction—A Comprehensive Review

**DOI:** 10.3390/jpm14040392

**Published:** 2024-04-08

**Authors:** Biagio Barone, Luigi Napolitano, Pasquale Reccia, Francesco Paolo Calace, Luigi De Luca, Michelangelo Olivetta, Marco Stizzo, Andrea Rubinacci, Giampiero Della Rosa, Arturo Lecce, Lorenzo Romano, Carmine Sciorio, Lorenzo Spirito, Gennaro Mattiello, Maria Giovanna Vastarella, Salvatore Papi, Armando Calogero, Filippo Varlese, Octavian Sabin Tataru, Matteo Ferro, Dario Del Biondo, Giorgio Napodano, Vincenzo Vastarella, Giuseppe Lucarelli, Raffaele Balsamo, Ferdinando Fusco, Felice Crocetto, Ugo Amicuzi

**Affiliations:** 1 Division of Urology, Department of Surgical Sciences, AORN Sant’Anna e San Sebastiano, 81100 Caserta, Italy; ferdinando.fusco@unicampania.it (F.F.); ugo.amicuzi@aorncaserta.it (U.A.); 2 Department of Neurosciences and Reproductive Sciences and Odontostomatology, University of Naples Federico II, 80131 Naples, Italy; luigi.napolitano@unina.it (L.N.); andrearubinacci1@gmail.com (A.R.); giampierodellarosa@gmail.com (G.D.R.); arturo.lecce92@gmail.com (A.L.); lorenzo.romano@unina.it (L.R.); gennaro.mattiello@unina.it (G.M.); salvatorepapi@blu.it (S.P.); felice.crocetto@unina.it (F.C.); 3 Urology Unit, AORN Ospedali dei Colli, Monaldi Hospital, 80131 Naples, Italy; pasquale.reccia@ospedalideicolli.it (P.R.); francescop.calace@ospedalideicolli.it (F.P.C.); raffaele.balsamo@ospedalideicolli.it (R.B.); 4 Division of Urology, Department of Surgical Multispecialty, AORN Antonio Cardarelli, 80131 Naples, Italy; luigideluca86@gmail.com; 5 Urology Unit, Gaetano Fucito Hospital, AOU San Giovanni di Dio e Ruggi d’Aragona, 84085 Mercato San Severino, Italy; m.olivetta@sangiovannieruggi.it; 6 Urology Unit, Department of Woman, Child and General and Specialized Surgery, University of Campania Luigi Vanvitelli, 80131 Naples, Italy; marco.stizzo@policliniconapoli.it (M.S.); lorenzospirito@msn.com (L.S.); 7 Urology Unit, ASST Ospedale Manzoni, 23900 Lecco, Italy; carmine.sciorio@gmail.com; 8 Gynaecology Unit, Department of Woman, Child and General and Specialized Surgery, University of Campania Luigi Vanvitelli, 80131 Naples, Italy; mariagiovannavastarella@hotmail.it; 9 Department of Advanced Biomedical Sciences, Section of General Surgery, University of Naples Federico II, 80131 Naples, Italy; armando.calogero2@unina.it (A.C.); filippo.varlese@unina.it (F.V.); 10 Department of Simulation Applied in Medicine, The Institution Organizing University Doctoral Studies (I.O.S.U.D.), George Emil Palade University of Medicine, Pharmacy, Sciences, and Technology from Târgu Mureș, 540142 Târgu Mureș, Romania; sabin.tataru@umfst.ro; 11 Department of Urology, European Institute of Oncology (IEO) IRCCS, 20141 Milan, Italy; matteo.ferro@ieo.it; 12 Department of Urology, Ospedale del Mare, ASL NA1 Centro, 80147 Naples, Italy; dario.delbiondo@aslnapoli1centro.it (D.D.B.); giorgio.napodano@aslnapoli1centro.it (G.N.); 13 Department of Translational Medical Sciences, University of Campania Luigi Vanvitelli, 80131 Naples, Italy; vincenzovastarella@hotmail.it; 14 Division of Cardiology, Cardiovascular Department, AORN Sant’Anna e San Sebastiano, 81100 Caserta, Italy; 15 Urology, Andrology and Kidney Transplantation Unit, Department of Emergency and Organ Transplantation, University of Bari, 70124 Bari, Italy; giuseppe.lucarelli@uniba.it

**Keywords:** bladder cancer, urinary diversion, ureterostomy, ileal conduit, orthotopic neobladder

## Abstract

Bladder cancer ranks as the 10th most prevalent cancer globally with an increasing incidence. Radical cystectomy combined with urinary diversion represents the standard treatment for muscle-invasive bladder cancer, offering a range of techniques tailored to patient factors. Overall, urinary diversions are divided into non-continent and continent. Among the first category, cutaneous ureterostomy and ileal conduit represent the most common procedures while in the second category, it could be possible to describe another subclassification which includes ureterosigmoidostomy, continent diversions requiring catheterization and orthotopic voiding pouches and neobladders. In this comprehensive review, urinary diversions are described in their technical aspects, providing a summary of almost all alternatives to urinary diversion post-radical cystectomy.

## 1. Introduction

Bladder cancer (BC) represents the 10th most common cancer in the world, with over 550,000 people diagnosed and 200,000 deaths related to this malignancy in 2018, according to GLOBOCAN data [1,2,3]. Up to 90% of BC cases arise from the urothelium while the remaining 10% of cases are represented by cancers arising from squamous cells or neuroendocrine cells [4]. Among the most recognized risk factors, male gender, tobacco smoke, age and occupational exposures represent the most common, albeit novel risk factors are currently emerging, such as red meat consumption, metabolic syndrome and interference of drugs [5,6,7,8,9]. Additionally, BC is accounted as one of the most expensive cancers to treat, with a cost of 150,000$ per capita for muscle-invasive bladder cancer (MIBC) [10]. The standard surgical treatment for MIBC is represented by radical cystoprostatectomy in male patients and anterior pelvic exenteration in female patients, along with en-bloc pelvic lymphadenectomy. The reconstruction of the lower urinary tract is required for the procedure, and it could be performed with, virtually, every segment of the gastrointestinal tract. Over time, urinary diversion following cystectomy has advanced significantly, progressing from a basic method of diverting urine to achieving almost complete functional restoration. The first reported urinary diversion following cystectomy was described by John Simon in 1852, which developed a urinary diversion via the rectum [11]. Successively, prior to 1950, the anal sphincter has been used for continence, establishing ureterosigmoidostomy as one of the urinary diversion of choice, until Bricker popularized, in 1950, the use of the ileum in the development of the urinary conduit, publishing a case series of 307 cases and describing a mortality rate of only 3.4% [12]. The ileal conduit by Bricker has not substantially changed from its publishing and it has been regarded, until recently, as the gold standard and predominant method for restoring the urinary tract. Currently, no ideal method of urinary diversion has been established. The recent advancements in urinary tract reconstruction have led to the identification of two broad categories: non-continent and continent urinary diversion. Among the latter, a further subcategorization could be evidenced, comprehending cutaneous reservoir and orthotopic neobladder [13]. Several factors influence the choice of urinary diversion, including patient age, body habitus, manual dexterity, overall health, mental well-being, kidney function, prognosis of the primary disease, pre-existing bowel conditions, prior exposure to radiation or chemotherapy and the presence of urethral disease. Additionally, exceptions, preferences, patient fears as well as the experience and preferences of surgeons, should be considered in the preoperative assessment when selecting urinary diversion methods. This comprehensive evaluation ensures a thorough understanding of the risks and benefits associated with each technique [14]. Furthermore, a well-informed decision regarding the type of urinary diversion correlates with decreased post-operative decision regret, independently from the chosen technique [15]. This comprehensive review aims to provide an overview of current technical alternatives in urinary diversions, starting from the simpler non-continent urinary diversions to the more complex and technically challenging orthotopic neobladders.

## 2. Non-Continent Urinary Diversion

Non-continent urinary diversions represent the most common type of reconstruction following radical cystectomy. Up to 91% of patients undergoing radical cystectomy received an ileal conduit from 1988 to 1999 while only 6.4% of patients received orthotopic neobladder [16]. Several factors may contribute to these findings, with patients’ age at the time of cystectomy likely being the most significant considering that the median age at diagnosis is 70 years and that elderly patients often experience general frailty. Non-continent urinary diversions, indeed, typically require a shorter operative time compared to continent reservoirs, leading to a reduced risk of postoperative complications or extended hospital stay. Additionally, a higher incidence of non-continent urinary diversions could be also attributed to advanced disease status at the time of surgery [17,18].

### 2.1. Cutaneous Ureterostomy

Cutaneous ureterostomy (UCN) was originally described as a method of urine diversion in children which was lately adapted for use in adults with ureteral obstruction related to malignancy. It stands as the most straightforward form of cutaneous diversion and presents lower operating time, blood loss and complications rate compared to the ileal conduit. This permits the use of UCN in frail patients or individuals with a solitary kidney requiring a supravescical diversion [19]. UCN could be made as a single ureterostomy on each side of the abdomen or in a double-barrel manner, permitting to obtaining a single stoma containing both ureters. The necessity to use an intrauretheral catheter to maintain patency is the main disadvantage due to the elevated incidence of urinary tract infections (UTIs). Considering the stomal stenosis rate superior to 50%, the UCN has limited its application and it is not recommended as a primary choice for palliative diversion considering the better results of percutaneous nephrostomy [20]. Along with stomal stenosis, the most frequent complication is the recurrent UTI which is associated with long-term renal dysfunction and calculi formation [21]. While rare, ureter-aortic fistula represents a fatal complication of UCN [22]. The relative simplicity of the procedure has permitted one to transpose this type of urinary diversion easily into laparoscopic and robot-assisted surgery [23,24].

### 2.2. Ileal Conduit

Ileal conduit represents the commonest urinary diversion method after radical cystectomy, accounting for 33% to 63% of cases [25]. Different approaches are reported in the formation of an ileal conduit. Generally, 15–20 cm of the distal ileal segment is isolated, 15 cm from the ileocecal valve. The isolated segment is inspected for its suitability, ensuring that the vascular arcade within the mesentery is neither under tension nor damaged. After performing the intestinal anastomosis, the ureters are mobilized and implanted on the proximal end, positioning the stoma usually below and right of the umbilicus [26]. For ureteral implantation, both end-to-side and double-barrel techniques are described by several authors. The ureter should be spatulated prior to anastomosing to the ileum [27,28]. The creation of an ileal conduit is technically more straightforward compared to a continent bladder substitution system but is more invasive compared to UCN. The advantage of the technique is the avoidance of intrauretheral catheters for ureters’ patency. Complications of ileal conduit are divided into early and late, according to the cut-off of 90 days after the procedure. Among the early complications included are those related to the bowel resection, i.e., obstruction, anastomotic leak and ileus, which could occur in up to 20% of cases. Among the delayed complications are reported parastomal hernia, retraction or stenosis and bleeding. UTIs, despite a lower rate compared to UCN, are similarly frequent in those patients [29,30]. In particular, up to 80% of patients report a deterioration of renal function which results in 6% of patients dying from end-stage renal failure [31]. Similarly to the UCN, ileal conduit has also been successfully early transposed to minimally invasive surgery [32,33].

### 2.3. Jejunal, Gastric and Colonic Conduit

Conduit utilizing other segments of the gastrointestinal tract has been once heralded as an alternative form of diversion for patients where the use of ileum was impractical due to prior irradiation, surgery or concurrent disease processes [34,35,36]. Despite the peculiar characteristics of these segments, such as the largest diameter of the jejunum, the poor absorbing mucosa of the stomach and the long mesentery of the colon, they are rarely used today due to the high rate of complications and electrolyte imbalance compared to the ileum [37,38,39]. The procedure related to the construction of these conduits is technically similar to the ileal conduit, with the ureters intubated with ureteral stents and anastomosed to the conduit according to Wallace (head-to-tail anastomosis) [40,41].

## 3. Continent Urinary Diversions

Continent urinary diversions involve the integration of segments of the both small and large intestine into the urinary tract. Reconstructive urology aims to recreate a normally functioning lower urinary tract for what concerns voiding, storage, continence and preservation of renal function. The continent urinary diversions allow patients to obtain an improved quality of life compared to non-continent urinary diversions. The ideal method of bladder reconstruction should include a non-refluxing mechanism, a low intraluminal pressure, provide adequate continence and be non-absorbitive [42]. Candidates for continent urinary diversions should undergo a thorough preoperative investigation and counseling, in addition to being appropriately informed regarding the potential complications associated with this type of reconstruction. A detailed history of the patient is required, with particular attention to previous abdominal/pelvic surgery, irradiation, intestinal resection, renal failure, diverticulitis and inflammatory bowel diseases. Absolute contraindications for continent urinary diversions are represented by compromised renal function, severe hepatic dysfunction and inflammatory bowel diseases while relative contraindications are represented by mental or manual impairments and previous abdominal or pelvic radiation [43]. Overall, continent urinary diversion could be classified into three categories: the first group involves ureterosigmoidostomy, which allows urine excretion through evacuation; the second group comprises continent diversions requiring catheterization to empty urine from the created reservoir; the third group consists of orthotopic voiding pouches [13,44].

### 3.1. Ureterosigmoidostomy

Ureterosigmoidostomy was, historically, among the first methods of urinary diversion widely utilized in patients with bladder exstrophy, lately applied to BC patients as a form of continent urinary diversions. Ureterosigmoidostomy necessitates a competent anal sphincter and a normally functioning sigmoid colon, in addition to a healthy renal function and little or absent ureteral dilatation. The surgical procedure consists of the insertion of ureters into the sigmoid colon in a non-refluxing manner. Overall, three groups of ureterocolic anastomoses are used: the tubular implantations with a submucosal tunnel (Coffey’s procedure); the ureterocolic implantation by direct end-to-side anastomosis; the tunneled direct end-to-side implantation. The first technique consists of an incision of the seromuscular layer of the colon for ureter insertion which is successively drawn into the intestinal lumen and fixed to the colon wall. The second technique consists of the spatulation of ureter ends which are anastomosed, with a single layer, to colon incisions. The third technique combines the previously described techniques providing a submucosal tunnel for ureters which are successively anastomosed edge-to-edge to the mucosal opening and covered by the seromuscular layer [45]. This enables patients to uphold continence and expel urine through the anus, eliminating the necessity for catheterizable abdominal stomas or cutaneous urinary diversion. Although the procedure has been widely used with excellent functional results reaching 80% of day and night continence, it has been abandoned due to the increased risk of metabolic abnormalities, infections, incontinence and secondary malignancy which may appear in up to 40–60% of patients [46,47]. In particular, the most common complications include hydronephrosis, pyelonephritis, anastomosis stenosis (often seen after Coffey’s procedure), coloureteral reflux, electrolyte imbalance and osteomalacia [48,49]. Due to these reasons and to the increased rate of colon cancer, which has been reported to be among the 2 and 15%, this technique has been mostly abandoned or conducted as a last resort in selected cases [50,51,52].

### 3.2. Kock Pouch

The Kock pouch is a continent ileostomy introduced by Nils Kock in 1964 and applied as a urinary diversion which could be intermittently evacuated utilizing a catheter. The pouch was designed to create an intrabdominal pouch made from the terminal ileum able to store the ileal content. Although the procedure may vary depending on the surgeons’ preference, the technique consists of the isolation of approximately 70 cm of small bowel segment, 50 cm proximal to the ileocecal valve, leaving 15 cm proximally and distally for creating the inlet and outlet of the pouch. The preparation of the nipple valve is performed stripping peritoneum and mesenteric fat off from the mesentery that supplies the intestinal segments intended for intussusception. The middle portion of the ileal segment is successively opened along its antimesenteric border and folded into a U-shape, suturing together the arms of the U which are 20 cm long each. The bowel wall is grasped through the opened lumen and the undivided ileal segments are partially intussuscepted into the lumen of the future reservoir, resulting in the formation of a 5 cm-long nipple valve. The intestinal plate is then folded up and the reservoir closed while the corners of the reservoir are pushed downwards between the mesentery, and the ureters are anastomosed to the proximal end of the inlet segment while the outlet segment is pulled through the channel in the abdominal wall to form the external stoma [53]. Albeit the appealing alternative to non-continent urinary diversions, the main drawbacks of the Kock pouch are the complex and lengthy procedure and the recurrent complications which occur in about 50% of patients and include pouchitis, enterocutaneous fistula and stricture [54].

### 3.3. Mainz Pouch

The Mainz Pouch procedure has been utilized since 1983 and has gone through several different phases of development. The original concept of a multipurpose ileocecal reservoir usable for bladder augmentation, orthotopic neobladder substitution or continent urinary diversion has remained unchanged. The procedure is based on the detubularization of an ileocecal segment, permitting for obtaining the advantages of the small and large intestine, utilizing the ileocecal valve as a reliable continence mechanism in the urinary diversion. The procedure involves the mobilization of the cecum and ascending colon up to the right colonic flexure with an excision of 10–15 cm of large bowel plus two loops of terminal ileum. After the spatulation of the ileum, adjusted to the bowel diameters, the bowel continuity is reestablished. In the case of a continent cutaneous diversion, the appendix stoma represents the first-choice continence mechanism, replacing the ileal intussusception nipple. The ileocecal segment is isolated and the tip of the appendix is resected and calibrated to 16–18 F in order to insert a similar measure catheter into the cecum via the appendix. After the antimesenteric splitting of the bowel and formation of the posterior wall, the pouch is completed after the implantation of ureters and closure of the anterior wall. The stump of the appendix is pulled through the abdominal wall and sutured on the skin. Other variants include modification of the pouch using a seromuscular bowel-flap tube or a full-thickness bowel-flap tube [55,56]. In addition to the classic Mainz pouch, two further variations have been developed: the Mainz pouch II, which uses a rectosigmoid pouch and the Mainz pouch III which instead uses 35–40 cm of ascending and transverse colon to shape the pouch [57,58]. The procedure has been also utilized successfully in minimally invasive surgery [59].

### 3.4. Cologne Pouch

The Cologne pouch is shaped utilizing the proximal sigmoid colon, incised at the level of the distal colon. An end-to-side anastomosis is formed between the descending colon and the rectum while the sigmoid is positioned into a U-shape and detubularized in order to create a cavity i.e., the sigmoid pouch. However, it has to be noted that this is a pouch which is mostly used for reconstructive urology in patients with bladder exstrophy-epispadias complex [50,60].

### 3.5. Penn Pouch

The Penn pouch was the first continent diversion employing the Mitrofanoff principle, i.e., the appendix used as a continence mechanism. Its use in the common clinical practice has been abandoned albeit representing a step forward to other complex and efficient pouches. Similarly to other described pouches, the technique presents two variants. The classical one, which includes the Mitrofanoff, consists of the excision of the appendix with a button of the cecum, reverted upon itself before proceeding to the tunneled implant [61]. Alternatively, the appendix could be left attached to the cecum and buried in an adjacent taenia. Successively the appendix tip is excised and grasped through to the selected stoma site [62,63]. Complications are the same as the previously described pouches [64,65].

### 3.6. Indiana Pouch

The Indiana pouch was developed by Mike Mitchell, Randall Rowland and Richard Bihrle at the Indiana University in the early 1980s considering the summative characteristics of the ileal conduit, Mitrofanoff principle and Kock Pouch [66]. The Indiana pouch is constructed utilizing the ascending colon and the terminal ileum, 15–20 cm away from the ileocecal valve. The isolated right colon segment is detubularized along the taenia coli and shaped into a U configuration. A pseudo-appendix is then formed utilizing a 12 F nelaton catheter introduced into the ileal segment, which is then tapered over using a stapler. The left ureter is crossed over to the right and passed below the mesosigma. A direct mucosa to mucosa anastomosis is performed, prior to a proper spatulation of the ureters. Successively, the ureteroenteric anastomoses are stented and brought out from the pouch through the abdominal wall. The rest of the pouch is then sutured in a spherical reservoir and the continence is tested before proceeding to the exteriorization and the fixation of the previously tapered ileum [67]. Despite the Indiana pouch presenting better quality of life outcomes compared to non-continent urinary diversions due to the presence of a catheterizable reservoir, the most commonly reported complications, in the long run, are stomal stenosis, infection, stones and leakage which were mostly related to the efferent limb [68,69]. Nevertheless, the Indiana pouch is still widely used, and long-term follow-ups are available in the literature, including also laparoscopic and robot-assisted approaches [70,71,72].

### 3.7. Florida Pouch

The Florida pouch was created by Lockhart et al. and it is formed from the cecum and the ascending colon. An intestinal segment consisting of approximately 10–12 cm of the distal ileum, cecum, ascending colon and right half of the transverse colon is isolated, folded and positioned in the right hemiabdomen. Subsequently, the colon is completely detubularized, exposing the bowel mucosa. The ureters are then brought into the open reservoir for reimplantation through a direct mucosa-to-mucosa anastomosis. Variations involving an antireflux mechanism such as a submucosal tunnel or utilization of the ileocecal valve, along with double plication of the efferent segment, have also been described. Ureteral stents are left indwelling and brought out through an opening into the abdominal wall [73]. Despite a fair diffusion in the nineties, the Florida pouch has been progressively abandoned and its use is currently very limited in selected cases where the small intestine is not usable [74].

### 3.8. Miami Pouch

The Miami was first described by Bejan and Rowland in 1981 and widely used in the early nineties and uses the same bowel segments as the Florida pouch, differing in the U-shape for the anti-mesenteric opening of the segments included [75,76]. The technique involves making an incision along the Toldts’s fascia in the right paracolic gutter and detaching the colon up to the first third of the transverse colon. This facilitates the removal of the right colon and the restoration of the intestinal continuity. The isolated intestinal segment extends from the colic angle to an ileal loop located 15 cm upstream of the ileocecal valve. Successively, the isolated ileocolic segment is detubularized and folded into a U shape along the antimesenteric side. The upper end of the pouch remains open until ureteral reimplantation is performed. A 14 F Foley catheter is then inserted into the pouch through the ileal loop to calibrate the efferent tube. Ureteral reimplantation is performed in a direct mucosa-mucosa manner, with a 1 cm prolapse inside the colon to allow a certain protection from urinary reflux. The pouch is then positioned according to the preoperative decision for the external orifice and the efferent segment is brought out to the abdomen wall and fixed to the skin [75]. Differently from the previously described Florida pouch, this technique is still widely used, due to the easier reproducibility and durability compared to other pouches. Additionally, the Miami pouch is regarded as a safer and more feasible alternative to orthotopic neobladder in patients not eligible for this type of urinary diversion [77,78,79].

### 3.9. Abol-Enein Pouch

The Abol-Enein pouch was described in 1999 by Abol-Enein and Ghoneim and consists of the identification and isolation of a 60 cm segment of terminal ileum which is divided into three parts. The middle 40 cm are configured in a W-shaped and utilized to construct the reservoir while the tapered proximal and distal 10 cm segments serve to prevent reflux and provide a continent outlet. Alternatively, if the appendix is available, it could be used as a continent outlet. Three to four mesenteric windows are created between the arterial arcades supplying proximal and distal segments. Each segment is successively inserted into its respective serous-lined trough and secured within the serous-lined extramural troughs. The spatulated distal ends of the tapered segments are anastomosed to the tunnel flaps while the ileal trough is closed in front of the embedded segment. The ureters are anastomosed to the proximal end of the inlet using a stented end-to-side mucosa-to-mucosa technique [80]. The peculiarity of this pouch is related to the antireflux mechanism built for the proximal and caudal segments which incorporates both a passive element, derived from the tubular resistance of the tapered ileal segment, and a dynamic element resulting from embedding within the reservoir wall [81].

## 4. Neobladders

The orthotopic neobladder allows voiding through the native urethra, eliminating the requirement for stoma appliances or catheters, while maintaining effective cancer control. The technique involving the creation of orthotopic neobladder has changed and evolved over time, incorporating modifications in shapes and approaches, such as the adoption of totally intracorporeal robotic-assisted techniques. Orthotopic neobladder permits to obtain an improved quality of life thanks to the keeping of the patient’s body image and near-normal voiding [82]. Additionally, compared to other types of urinary diversion, improved post-operative sexual function and urinary continence are maintained [83]. The selection of a patient for an orthotopic neobladder reconstruction is however stricter compared to the other urinary diversions as this type of reconstruction requires, in addition to a healthy intestine and renal function, mental and physical competency in order to allow appropriate neobladder training post-procedure [84]. The procedure requires the reconstruction of intestinal segments, leading to two main considerations. The first is the availability of the terminal ileum, which is the most favoured intestinal segment used for reconstruction, due to its distensibility and larger capacity, permitting it to store urine at lower pressures. Additionally, the terminal ileum is subject to more mucosal atrophy in the long term compared to other segments, reducing metabolic consequences related to electrolyte exchange via the mucosa [85]. The second consideration is bound to the type of construction, considering that the natural cylindrical shape of intestinal segments would result, according to the Laplace law (intraluminal pressure in a hollow tube is inversely proportional to its radius), in an intolerable high intraluminal pressure. Due to these premises, the bowel is detubularized in order to permit the refashion of the selected segment into a proper reservoir, obtaining a larger radius and lower intraluminal pressures [86]. This permits indeed to deny the complete transmission of myogenic activity from the longitudinal muscle to the inner circular muscle, reducing contractions and intraluminal pressures [87]. According to these considerations, the best shape for an orthotopic neobladder would be the sphere or the ellipse due to the smallest surface area for the same volume, requiring the minimal length of the intestinal segment while maintaining an adequate capacity with the smallest risk of metabolic consequences related to the surface exposed to urine [88,89]. The widespread adoption of laparoscopic and robot-assisted techniques has permitted the diffusion of orthotopic neobladders which were once considered complex and technically demanding procedures. The utilization of small incisions, precise instrumentation and minimal manipulation of tissues and organs have resulted in shorter hospital stays, lower risk of complications and quicker recovery of patients [90,91]. Currently, all the following techniques, mostly developed during the pre-minimally invasive surgery era, have been adapted successfully to laparoscopy and robot-assisted surgery up to the totally intracorporeal reconstruction of the lower urinary tract [92,93].

### 4.1. Studer Neobladder

**Figure 1:**
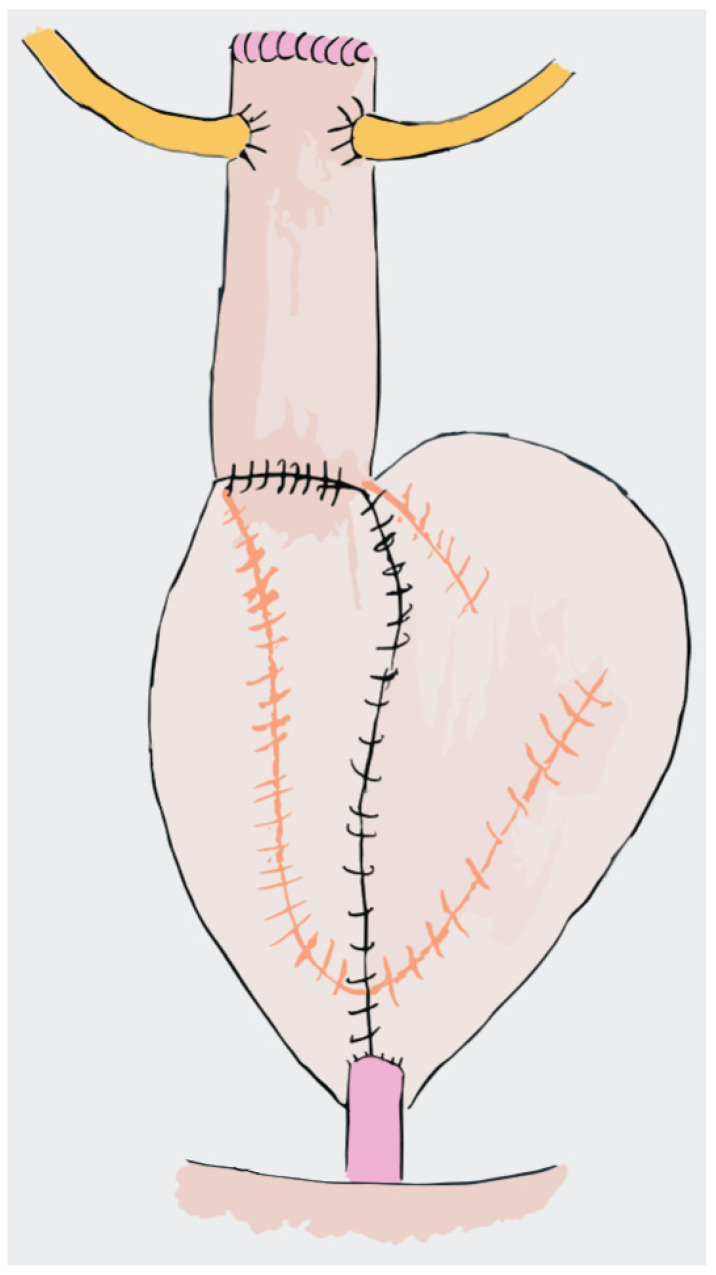
Studer Neobladder.

The Studer neobladder was first published in 1989 and has become a common approach for orthotopic neobladder reconstruction [94]. In 2011, an intracorporeal version of the Studer neobladder was also described [95]. The technique is performed via the isolation of an ileal segment of 50–60 cm, 25 cm proximally to the ileocecal valve. The ileourethral anastomosis is performed initially, followed by isolating a segment of the ileum extending 40 cm proximal and 10 cm distal to the anastomosis. Ten centimetres away from the proximal end, which is reserved for the chimney, the ileum segment is opened on its antimesenteric border and three stay sutures are placed to aid bowel segment positioning. The posterior plate of the neobladder is then closed while the lateral limb is folded over to create a spherical neobladder. Lastly, the distal part of the anterior neobladder is closed and a Wallace ureteroileal anastomosis is performed at the proximal end of the previously cited chimney, before completing the closure of the anterior plate [96]. The advantage of the Studer neobladder is the relative simplicity of the technique and the possibility of moving the reservoir fairly down to the urethra, which proved to be a reproducible procedure with good operative and functional outcomes [96] (Figure 1).

### 4.2. Hautmann Neobladder

**Figure 2:**
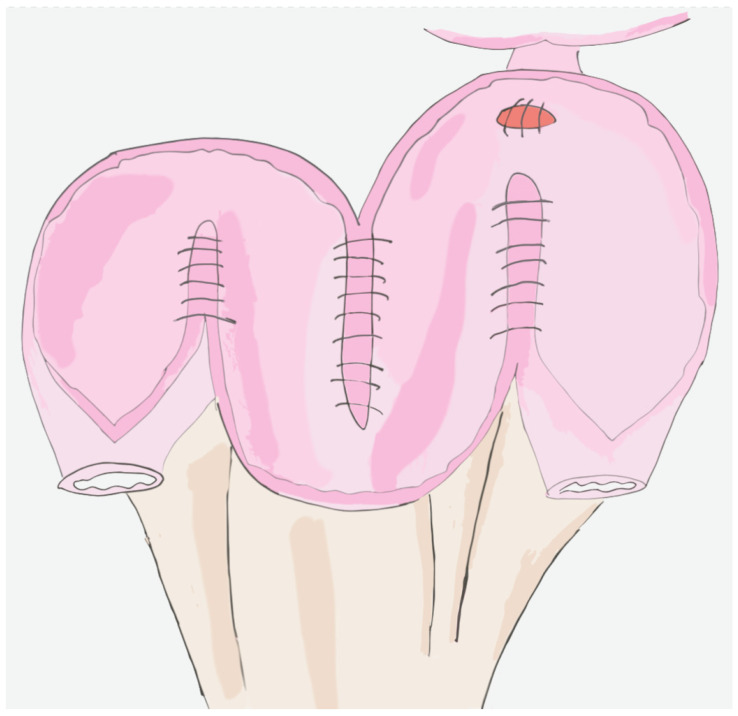
Hautmann Neobladder.

The classic Hautmann neobladder is performed by selecting and isolating 60 cm of terminal ileum which is then divided 20–25 cm proximally to the ileocecal valve. The isolated bowel segment is then incised on the antimesenteric side, with the exception of 2–3 cm short chimneys on both sides and the intended site of the ileourethral anastomosis. Four lengths of the ileum are shaped into a “W” with the previously described chimneys on each side. A buttonhole of all layers, 2–3 cm from the tip of the U-shaped flap. An ileal plate is then formed from the “W”, sewing together the edges on the antimesenteric border. A Foley catheter is placed through the buttonhole of the ileal plate and the anastomosis with the urethra is performed. The ureters are spatulated and anastomosed to the chimneys with the Wallace technique, in an open end-to-side fashion, with the ureters stented [97]. The neobladder is then positioned in the small pelvis, extraperitoneally. The remaining anterior neobladder plate is then closed in a T-shape [97]. Despite various modifications that have been reported in the literature, the Hautmann neobladder is, similar to the Studer neobladder, a technically simple and safe method for bladder replacement, with satisfactory results in terms of functional outcomes [98,99,100]. An intracorporeal version of the Hautmann neobladder, called W-shaped neobladder, has also been successfully performed [101] (Figure 2).

### 4.3. Kock Ileal Neobladder

The Kock ileal neobladder, described first in 1982, is based on the Kock pouch and recalls the same surgical technique, presenting two limbs of 17 cm, isolated 20 cm proximally to the ileocecal valve with a reservoir shaped in a spherical manner, utilizing 44 cm of small bowel [102,103].

### 4.4. Padua Ileal Neobladder

**Figure 3:**
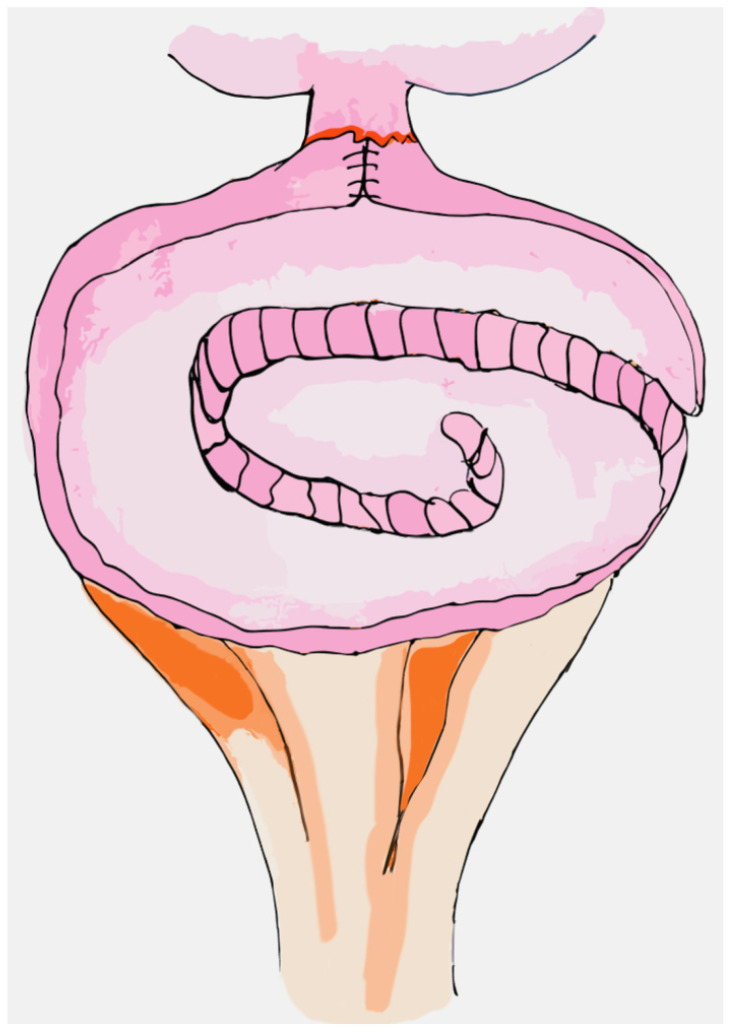
VIP Neobladder.

The Padua Ileal neobladder, also known as Vescica Ileale Padovana (VIP), is based on the resection of a 40 cm ileal segment, isolated 15–20 from the ileocecal valve. The distal loop, measuring about 20 cm, is modeled into a U-shape and the ileourethral anastomosis is performed on the inferior plate of this segment. The ileal segment is detubularized along the antimesenteric border and the proximal loop is folded medially on itself in order to shape a reversed U while the inner margins are sutured laterally to model a spherical reservoir. Ureters are then anastomosed on the posterior side of this spherical reservoir [104]. The technique, first described in 1987, is widely used in reconstructive urological surgery. To date, the VIP has also been performed in a totally intracorporeal manner during robotic-assisted surgery and represents one of the most common types of neobladder reconstruction [92,105,106,107] (Figure 3).

### 4.5. Y-Shaped Neobladder

The Y-shaped neobladder was described by Fontana et al. in 2004 and is one of the first to introduce the use of a mechanical stapler in order in the neobladder shaping process. The technique is performed by isolating 40 cm of the distal ileum, 15–20 cm proximally to the ileocecal valve. The isolated intestinal tract is successively folded and shaped in a Y, with the central segments measuring 14 cm and the arms of the Y measuring 6 cm. The central segments, juxtaposed, are then detubularized using a mechanical stapler inserted into the base of the Y. This base is successively anastomosed with the native urethra while ureters are sutured into the two arms of the Y. The relative simplicity of the technique and its good results of this reservoir in terms of outcomes and complications have permitted a wide use of this reconstruction, even in difficult cases [108,109]. The technique has also been utilized successfully in laparoscopic and robotic-assisted surgery and has been adapted in particular cases according to the characteristics of patients [110,111,112].

### 4.6. Camey I-II Neobladder

**Figure 4:**
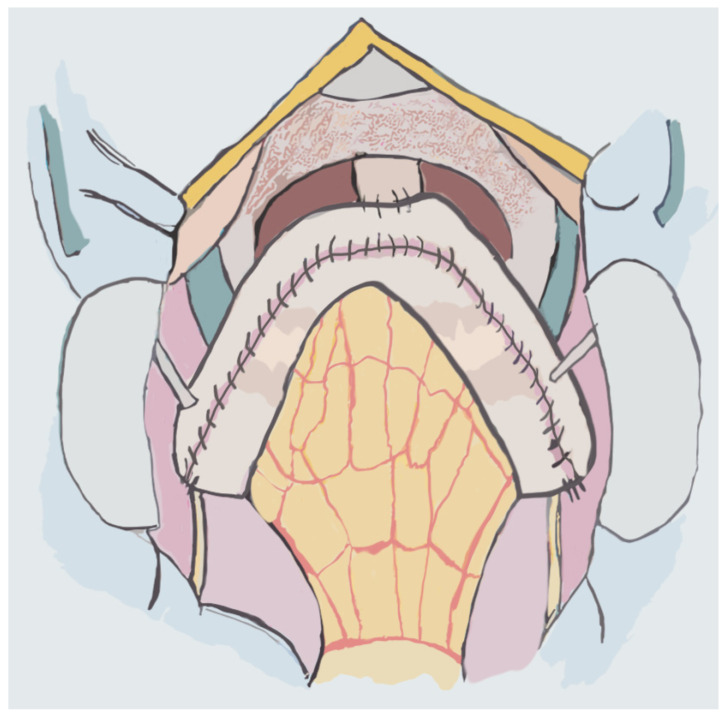
Camey Neobladder.

The Camey I Neobladder is built via the identification and isolation of a 40 cm segment of ileum which, modeled into a U, allows the apex to reach the native urethra in a tension-free manner. After performing the intestinal section and re-establishing its continuity, both extremities of the U are opened on the anti-mesenteric side. Next, 1.5–2 cm from the extremities, ileoureteral anastomosis is performed in an anti-reflux manner while the ileourethral anastomosis is performed by cutting 1–1.5 cm of ileum at the apex of the U [113] (Figure 4).

The Camey II neobladder is built via the identification and isolation of a 70 cm segment of ileum which is opened on the anti-mesenteric, laterally juxtaposed and then sutured via mechanical stapler in order to model a neobladder similarly to Camey I. Differently from the latter, the Camey II presents a greater surface available for constructing the reservoir. Ileourethral and ileoureteral anastomoses are performed similarly to the previously described Camey I [114,115].

### 4.7. U-Shaped Neobladder

The U-shaped Neobladder includes the use of a mechanical stapler and the presence of two arms. A total of 40 cm of the ileum are isolated, 15–20 cm from the ileocecal valve. A side-to-side anastomosis permits reestablishing intestinal continuity and obtaining an intestinal segment to shape into a U, with two arms of 20 cm and an afferent portion of 5–10 cm. In the distal portion of the U, an incision on the anti-mesenteric side is performed in order to allow the insertion of the mechanical stapler and finish the detubularization of the segment while permitting the fusion of both arms of the U. Ureters are then anastomosed, respectively, into the distal portion of the two arms while the apex of the U is used for the ileourethral anastomosis [116]. The technique, born for laparoscopic surgery, has been successively and successfully utilized in robotic surgery [117].

### 4.8. Z-Shaped Neobladder

The Z-shaped Neobladder uses an intestinal segment of 45 cm, proximally isolated 20 cm from the ileocecal valve. Once intestinal continuity is re-established, the chosen ileal segment is shaped into a Z and opened along the anti-mesenteric side. The inferior parts of the Z form a cup-like reservoir while the upper part of the Z acts as a cover of this cup. In the inferior part, ileourethral anastomosis is performed while ureters are anastomosed upper and posteriorly [118]. Despite the good results in terms of complications and continence, under 500 cases are reported in the literature, including slight variations of the technique [119].

### 4.9. Anatolian Ileal Neobladder

**Figure 5:**
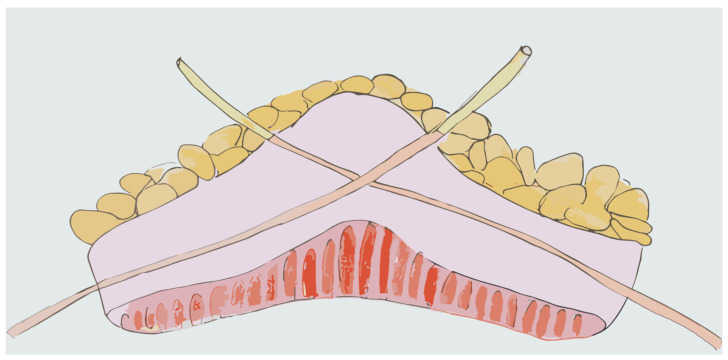
Anatolian Neobladder.

The Anatolian Ileal Neobladder, described by Talat et al. in 2018, includes an ileal segment of 45 cm which is isolated 15–20 cm from the ileocecal valve and successively opened on the anti-mesenteric side. The proximal and distal sides of the ileal segment are anastomosed side-to-side and successively modeled into a donut-shape. Three points are identified on the medial side of the anastomosis and joined centrally while the lateral sides of the loop are shaped into a triangle. Ileoureteral and ileourethral anastomoses are then performed on the apex of this triangle [120] (Figure 5).

### 4.10. Shell Neobladder

The Shell Neobladder has been developed for totally intracorporeal neobladder reconstruction in robotic surgery. A total of 40 cm of the ileum are identified 15 cm from the ileocecal valve. After the re-establishment of the intestinal continuity, the ileourethral anastomosis is performed at 20 cm from the selected ileal segment. Successively, the ileal segment is detubularized, and the posterior plate is reinforced with multiple sutures. A total of 10 cm of the anterior plate is then sutured in order to shape the neck of the neobladder while ureters are anastomosed posteriorly. Finally, the posterior plate is folded anteriorly, in a shell-like manner, and sutured to the anterior plate [121].

### 4.11. Florin Neobladder

**Figure 6:**
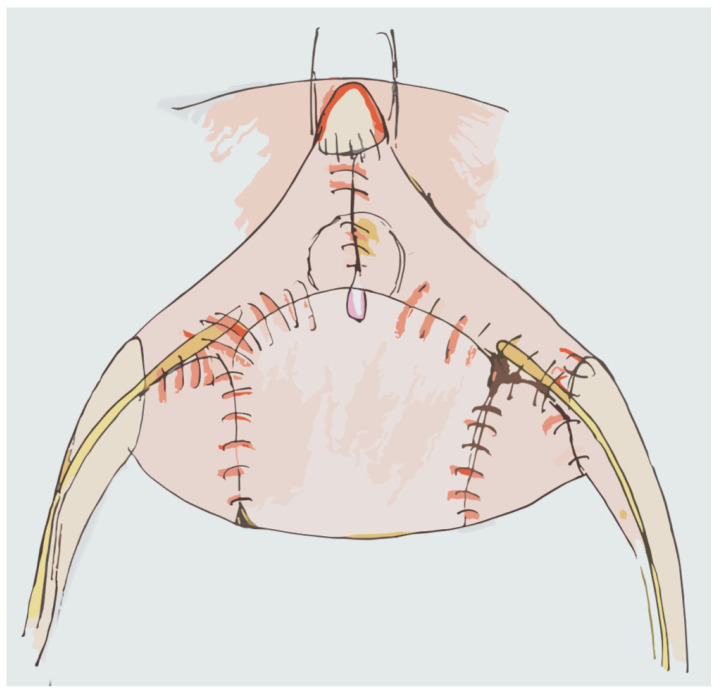
Florin Neobladder.

The Florin Neobladder has been developed for totally intracorporeal robotic surgery. After the identification of the cecum and the placement of a stay suture 20 cm from the ileocecal valve, 50 cm of the ileal segment is selected in a manner that permits it to reach the native urethra without tension. After the reestablishment of intestinal continuity, the ileourethral anastomosis is performed on the apex of an asymmetrical U, 30 cm and 20 cm from the anastomosis. The entire segment is systematically detubularized, and the posterior plate is reshaped into an L-shape to broaden the posterior support for the neobladder and form a neo-trigon. Following the construction of the posterior plate, the neobladder neck is reconstructed and the posterior plate is folded anteriorly, 5 cm from the proximal border of the posterior closure, thereby reconstructing the two asymmetric segments. The ileoureteral anastomoses are carried out laterally on both anterior segments. Subsequently, this anterior plate is closed into an inverted “V” shape [122] (Figure 6). Interestingly, the technique has also been replicated successfully laparoscopically, increasing the feasibility and the diffusion of this approach [123].

### 4.12. Vesuvian Orthotopic Neobladder

The Vesuvian Orthotopic Neobladder (VON), has been developed as a way to facilitate orthotopic neobladder reconstruction after radical cystectomy via the utilization of a mechanical stapler. A total of 36 cm of the ileum are identified and isolated about 15–20 cm from the ileocecal valve. Successively, after the selection of the intestinal loop, a side-to-side anastomosis is performed with a mechanical stapler in order to restore the intestinal continuity. The isolated intestinal loop is successively shaped into a three-horned triangle which permits the anastomosis with the urethra and the ureteral stumps. The mechanical stapler is used to permit a complete detubularization of the intestinal loop without excessive manipulation of the ileum [124]. The technique could also be performed in a totally intracorporeal manner [125].

## 5. Discussion

Urinary diversion after cystectomy has radically changed the treatment of malignant disease of the urinary tract since its conception over 150 years ago, evolving, from a basic diversion of the ureters to the skin to almost complete restoration of the previous bladder functionality [126]. As emphasized by the European Association of Urology (EAU) guidelines, radical cystectomy combined with urinary diversion represents the recommended standard treatment for non-metastatic MIBC while it is also presented as an advisable option for high-risk non-muscle-invasive bladder cancer (NMIBC) [127]. The different choices of urinary diversions after radical cystectomy are tailored according to the patient’s preference, performance status, life expectancy as well as oncological control. The ileal conduit is currently the most adopted reconstruction following radical cystectomy due to the relative ease of its formation and the shorter operative time, permitting its use in patients with significant medical comorbidities [128]. Albeit these advantages, early and late complications may occur, accounting for infective, gastrointestinal and wound complications among the most commonly observed events in the early postoperative period [129]. Additionally, the necessity of an external stoma with a urine collection bag could greatly impair the quality of life of patients with this type of urinary diversion [130]. Late complications include instead urolithiasis, renal morphological or functional changes, stomal and ureteral anastomotic complications. In particular, stomal complications represent a unique consideration in ileal conduit diversions, with 30–50% of patients developing parastoma hernias [131,132]. Similar issues are related to the construction of ureterocutaneostomy which, if on one side permits to avoid the creation of a bowel diversion, potentially reducing the risk of metabolic disturbances and complications related to bowel manipulation, on the other side is considered less continent than an ileal conduit, leading to skin irritation due to urine leakage on the site of ureterocutaneoustomy and requiring ureteral catheters in order to maintain patency of the ureters [133]. The renal deterioration is, further, faster than other types of diversions, advising the use of this diversion to patients with limited life expectancy or with multiple comorbidities impeding most complex reconstructive procedures [134,135]. Another option in the spectrum of urinary diversion is the creation of pouches, i.e., catheterizable reservoirs. Pouches permit obtaining a more physiological and continent means of storing and eliminating urine, avoiding the necessity of external stoma. Additionally, it could be possible to further hide the catheterizable conduit via the strategic position of the reservoir, thus further improving the body image and the quality of life [136,137]. The advantages of pouches include indeed the improved urinary continence and a more natural voiding pattern. Conversely, these procedures are technically more complex and require longer operative time with an increasing risk of complications, which are mostly related to urinary leakage, metabolic imbalances and long-term concerns such as pouchitis or secondary malignancy [69,138,139]. Additionally, careful patient selection and counseling are crucial considering that not all patients are suitable candidates for pouch reconstruction due to factors such as cognitive function, manual dexterity and willingness to comply with self-catheterization or voiding schedules [140]. Nevertheless, among the range of urinary diversion techniques available, the orthotopic neobladder stands out as the preferred choice among patients undergoing radical cystectomy due to its ability to maintain a higher quality of life when compared to alternative forms of urinary diversion [141]. This preference is attributed to the absence of a stoma, enabling natural voiding through the urethra, which aligns better with social acceptance [142]. However, the construction of a functional neobladder following radical cystectomy remains a formidable and technically intricate procedure within the urology field, regardless of the chosen approach. Due to this reason, the utilization of orthotopic diversion in the common clinical practice has decreased over time [143,144]. Additionally, the possibility of offering a urinary diversion rather than another also depends on the comorbidities, the age and the oncological status of the patient. Indeed, orthotopic neobladder is mostly offered to younger and male patients with an early T stage [145]. As a result, a large variation has been described regarding the use of urinary diversion techniques with higher-volume centers of excellence reporting a greater prevalence of orthotopic neobladder usage. Conversely, several population-based datasets alternatively report up to 90% of the use of urinary conduits [141]. It is well known that the reason for this variation is to be found in the multifactorial evaluation of the technique of reconstruction to be used, considering that oncologic, functional and quality of life outcomes are involved, in addition to, as previously reported, other several characteristics related to the patient (kidney and liver function, dexterity, age, comorbidities) [84]. Determining the optimal urinary tract reconstruction is still a challenge for the urologist as no single technique is ideal for all patients and situations. Additionally, another variable influencing the type of reconstruction is related to the experience of the surgeons involved in the urinary diversion reconstruction, considering that the orthotopic neobladder is an intrinsically more complex procedure compared to other types of non-continent urinary diversions. Historically, most surgeons have favored extracorporeal methods for urinary diversion, particularly for neobladder reconstruction, due to extended operation times and the steep learning curve associated with intracorporeal techniques. Nonetheless, the increasing proficiency in robotic pelvic surgery, facilitated suturing with wristed instruments, improved ergonomics, enhanced visualization, and reduced risks of bowel manipulation have all contributed to sustaining the appeal of totally intracorporeal bladder reconstruction [146]. In terms of the ideal neobladder characteristics, existing literature underscores the importance of appropriate capacity, low-pressure storage, absence of reflux, and high compliance in order to promote continence while enabling voluntary voiding. The spherical configuration of the reservoir has been acknowledged as a favorable choice due to its capacity to hold larger volumes at lower pressures while also addressing concerns regarding electrolyte exchange [147]. However, it is important to note that an excessively large initial volume might not necessarily improve continence rates and could lead to progressive enlargement, resulting in atony and voiding difficulties [148].

Despite the advantages of the orthotopic neobladder, apart from surgical challenges, it has to be noted that complications of radical cystectomy and successive urinary diversion, are diffused and potentially life-threatening [149]. Among the early complications, bleeding, thrombotic events, infections and cardiopulmonary complications do not seem to differ among patients undergoing different types of urinary diversions. However, gastrointestinal complications are commonly associated with the intestinal manipulation needed for the construction of orthotopic neobladders or pouches [150]. Similarly, leakage and ureteral complications are unrelated to the type of diversion [151]. It should be always considered that radical cystectomy with urinary diversion represents a complex procedure and is characterized by a potentially high morbidity, such as those related to other major surgeries [152]. To this regard, the Enhanced Recovery After Surgery (ERAS) protocol has been implemented in order to improve perioperative outcomes and reduce the possibility of the aforementioned complications. The ERAS protocol is a multimodal perioperative care pathway designed to optimize patient outcomes following surgery and encompasses a series of evidence-based interventions aimed at reducing surgical stress, enhancing postoperative recovery and reducing complications [153]. Among the key elements of this protocol, preoperative patient education, nutritional optimization, minimization of preoperative fasting, early mobilization and reduction of opioid consumption, represent the most important measures to increase patient’s recovery. As for other major surgeries, the implementation of the ERAS protocol in radical cystectomy with urinary diversion has led to reduced intraoperative blood transfusion rate as well as overall length of stay [154,155]. Beneficial outcomes were also reported for the time to first flatus, gastrointestinal recovery and rate of 30-day readmission [156]. Among the late complications of orthotopic urinary diversion, which are directly related to the diversion itself include incontinence, urinary tract infection, urethral or ureteral strictures and stones [157,158]. Particular attention should be lastly given to the evaluation of continence and clinical outcomes related to continent urinary diversion. Indeed, the prevalence and severity of urinary incontinence may be influenced by several variables which include age, gender, prior treatments, surgical techniques and surgeon experience. Additionally, the subjective methods used to asses urinary continence represent another critical point, proposing, a certain caution in comparing continence results from different series of patients with orthotopic neobladder [159,160,161].

## 6. Conclusions

The evolution of urinary diversion techniques after cystectomy has significantly transformed the landscape of treating malignant diseases of the urinary tract over the past 150 years. The choice of urinary diversion following radical cystectomy remains dependent on surgeon preferences, experience, and technical feasibility, all aimed at achieving the optimal reservoir. The determination of the optimal urinary tract reconstruction remains a challenge and every technique has to be tailored and discussed with the patient, according to comorbidities and situations. The decision-making process should involve a comprehensive assessment of an individual patient’s characteristics, surgeon expertise and the goals of achieving oncologic control, functional outcomes and improved quality of life. Advances in surgical techniques and ongoing research are likely to contribute further to refine the choice of urinary diversion methods in the future.

## Data Availability

Not applicable, no new data were created.
